# Material Databases and Validation in Modelling the Structure of Castings Using the Cellular Automaton Method

**DOI:** 10.3390/ma14113055

**Published:** 2021-06-03

**Authors:** Jakub Hajkowski, Robert Sika, Michał Rogalewicz, Paweł Popielarski, Waldemar Matysiak, Stanislaw Legutko

**Affiliations:** 1 Division of Foundry and Plastic Working, Institute of Materials Technology, Faculty of Mechanical Engineering, Poznan University of Technology, 3 Piotrowo St., 60-965 Poznan, Poland; robert.sika@put.poznan.pl (R.S.); Pawel.Popielarski@put.poznan.pl (P.P.); waldemar.matysiak@put.poznan.pl (W.M.); 2 Division of Production Engineering, Institute of Materials Technology, Faculty of Mechanical Engineering, Poznan University of Technology, 3 Piotrowo St., 60-965 Poznan, Poland; michal.rogalewicz@put.poznan.pl; 3 Division of Technology Planning, Institute of Mechanical Technology, Faculty of Mechanical Engineering, Poznan University of Technology, 3 Piotrowo St., 60-965 Poznan, Poland; stanislaw.legutko@put.poznan.pl

**Keywords:** micromodeling, microstructure, cellular automaton, database, aluminum alloys

## Abstract

The paper presents the scope of applicability and the usefulness of the method of predicting crystalline structure of castings using a commercially available system called Calcosoft CAFE. The influence of individual values of the parameters of the thermal model and the model predicting the structure (phenomenon of nucleation and crystal growth), and the method of interpretation of the results were identified. In simulation studies, it is important to use reliable and validated material database, under appropriate conditions. It is necessary to predict the properties of castings with a comprehensive, new and practical approach to modelling the formation of phase components of structure in terms of both macroscale and microscale phenomena (Multiscale and Multiphysics). Therefore, in this paper, the experimental-simulation validation of the CAFE code was undertaken. The tests were carried out on castings solidifying under various heat transfer conditions controlled by mould materials such as: a homogenous mould made of moulding sand, moulding sand with chill, and mould made of insulating mass with chill. These conditions directly influence the structure formation. The method of validation of the structure was determined in terms of its three parameters, i.e., the degree of refinement of the crystals, the location of the columnar-to-equiaxed transition zone—CET and the angle of the crystals. The above tests enabled to extend the content of databases, which often lack the necessary values of parameters used in modelling, e.g., crystallization of a specific alloy under given conditions (sand casting, chills or laser surface treatment). On this basis, the basics of correlating the simulation results on a micro- and macroscale were generalized, the limits of the application of individual parameters (mould, alloy materials) and their impact on the structure formation were determined. It resulted in the extension of the database for simulation calculations.

## 1. Introduction

The development of computer techniques and IT science in connection with the broadening of knowledge about casting processes has led to great progress in the field of simulation of technological processes in the cast-mould system [1,2]. Virtualization plays a steadily increasing role in the design of mechanical engineering technologies.

Currently, there are few scientific and technical fields that do not use the term “modelling”, which means integration with numerical simulation. For the last thirty years, there has been an increasing number of publications, reviews and studies dealing only with these issues [1,2,3,4,5,6]. They can be found in the fields of solid mechanics, fluid mechanics, heat transfer, calculations of material structure formation, stress development, etc. The studies in which modelling and computer support have a dimension integrated with a real process, even if they describe complex particle phenomena, have the greatest value for foundry technology, otherwise, the scientific works are detached from the context of the application, which makes them hardly or completely not useful as supporting computer codes in foundry studies.

The main purpose of using simulation in casting processes with models in their basic coupled configuration (Navier-Stokes and Fourier-Kirchhoff) is to predict discontinuities in the casting as precisely as possible. This mainly concerns shrinkage defects (voids, porosity) [7,8], the level of acceptability of which is set by the customer in the acceptance conditions, in accordance with the principles of the “tolerance of damage” approach. It is confirmed by results of an inspection using non-destructive testing (NDT) methods, commonly used in loss-free technologies [9]. Still, despite the passage of many years of operation of these two basic model configurations of simulation systems and experience in their application, more perfect prediction algorithms are still being sought [1,10,11,12]. These algorithms are assigned to the dynamics of solid phase growth and the flows between the elementary volumes of the casting, ascribed to the elements of the discrete division of the casting. These flows are known to be caused by shrinkage phenomena during solidification and the related local demand for liquid/solid-liquid metal. The balance of demand, local flows and grain nucleation and growth phenomena are modelled differently by individual simulation system creators [1,2,3,4,5,12,13,14,15,16,17,18,19,20,21,22,23,24,25,26,27,28,29,30].

It is crucial in the simulation of the phenomena forming the crystallization and solidification of the alloy to predict not only these phenomena on a macroscale resulting from the variability of temperature fields, but also to extend this prediction with a microstructure and segregation of the solute [26,28]. By introducing micromodelling algorithms (modules) from the Multiscale and Multiphysics family, it is possible to simulate the formation of primary grains and even the texture (columnar, equiaxed) of dendrites. In this method utilized for the crystallization model, the physical phenomena of nuclei formation (nucleation process) and their growth must come to the fore. An obvious advantage of this approach is that it represents a more realistic model approach to the local (grain scale) release of latent solidification heat depending on the local undercooling of the liquid alloy [13,19,25,26], providing an opportunity for a better virtual look at the phenomena and comparing the results with the actual structure parameters.

Predicting the structure can be conducted with the use of simulation systems by means of coupled hard and soft modelling approaches. Hard models, i.e., those based on physical laws and the differential equations describing them, do not offer a satisfactory approach to a phenomenon as complex as the crystallization process. Therefore, it is necessary to look for solutions in the area of modelling in Multiscale and Multiphysics [1,2,6,16]. Systems of this type include the issues of several physical and/or physicochemical phenomena within one coupled model taking into account the discrete division scale, e.g., the most obvious in material technologies is macro-micro modelling. The aim is to try to reflect the real process as comprehensively as possible. However, this creates new validation tasks that must be undertaken in parallel with the creation of models intended for coupling. The inclusion of soft models seems to be indispensable for the “Multiscale” modelling problem to find solutions useful in foundry practice.

Therefore, in this work, for the experimental and simulation validation, the coupled macro-micro code Calcosoft— Cellular Automaton Finite Element (CAFE) (Ecole Politechnique Federale de Lausanne EPFL, Lausanne, Switzerland and ESI Group, Paris, France) [1,13,16,19,22,23,31,32] was applied, which is considered to be one of the best simulation codes for predicting the formation of columnar and equiaxed crystals in castings. This method has been developed since 1990 and consists in the primary discretisation of the casting by the finite element (FE) method—(heat transfer modelling—macromodel) and the secondary discretization of the modelled solid-liquid phase space, with the formation of cells with very small dimensions, of the value of 10^−4^–10^−6^ m (modelling of structure formation phenomena using a cellular automaton—CA—micromodel). Hence, this method was designated by its creators from Ecole Politechnique Federale de Lausanne—EPFL with the acronym CAFE [21,23].

## 2. State of the Art

According to Rappaz [13], the modelling scales can be formally divided into four groups, referring to four generations of simulation (modelling) systems covering the issues of crystallization and solidification of castings: macro-, meso-, micro-, and nano- [17,18,19,20].

Unfortunately, most of the described models from the Multiphysics family [1,2,14,15,27] in terms of predicting the structure formation refer to the local modelling of phenomena for very small, separated areas, without and reference to the entire casting and mould. For example, the growth of only single equiaxed crystals or at most a group of several crystals (dendrites) is generated, mostly often in two-dimensional way, without reference to the real process conditions, which is a necessary condition for practitioners and industrial realities [16]. The Calcosoft CAFE system tries to indicate the application direction in order to meet not only simple castings, but also those of a more complex shape.

Modelling of the structure of castings using the available simulation codes from the Multiscale/Multiphysics family is limited to single grains [1,4,15,18,29,30,33,34,35,36,37,38,39], e.g., in the form of a developed dendrite shape in 2D and 3-D space (dimension degree of the task influences on total cells number and computation time), or in the form of its simplified shape—primary tips inscribed into the structure of entire castings [22,23,24]. However, it includes the coupling of the macro- and microscopic levels. The macro level is related to the leading phenomenon—heat flow—while the micro level is related to the phenomena associated to the mechanisms of nucleation and growth of the grains (taking into account, for example, local distribution of the solute), and as a result generating the latent heat of solidification in proportions relevant to the grain volume increase. This enables to determine parameters such as: DAS1—the distance between the axes of the primary dendrites (because cellular automata, CA, do not allow modelling the growth of secondary dendrite tips by generating pseudo-dendrites in the form of crystallite blocks with the accuracy to the CA cell size), the size of the crystallites, the location of the columnar-to-equiaxed transition zone (CET).

In [24,37] the cellular automaton method has been coupled with the finite difference method to solve the phenomena of solute diffusion. Lee et al. [24,37] and Nastac [40] added a more precise technique—front tracking with the release of the solid phase in each growing cell, which allows to distinguish not only the outline, but also the morphology of the dendrite taking into account higher-order tips (arms).

Another approach (using the possibilities offered by the algorithms of numerical solutions in relation to analytical solutions) is to the estimate the transition limit of columnar dendrites into equiaxed forms as presented in the paper [18,29]. The coupled model is based on the Fourier-Kirchhoff equation and on the diffusion equation for three areas: the solid phase, liquid phase in the space between the dendrite tips, and the liquid phase outside the dendrite; and on the equations describing nucleation (according to the hypothesis developed by Stefanescu [18] and the growth of dendrites according to models: Kurz—the growth of the dendrite tip, Hunt—the space between the first-order arms, Kurz and Fisher—the distance between the second-order arms).

Martorano et al. [41] proposed a different hypothesis for the mechanism of a columnar-to-equiaxed transition zone (CET). The indicated model of directional crystallization is characterized by a certain temperature gradient. Equiaxed dendrites nucleate in the area below the temperature T_N_, lower than the liquidus isotherm (T_L_(C_0_)) in an undercooled liquid phase with a concentration range of C above C_0_. According to the classical theory of the phenomenon, equiaxed dendrites growing before the columnar dendrites enrich the liquid phase with an solute “pushed” beyond the crystal, which obviously causes an increase in the concentration of the liquid phase C_l_ above the initial concentration C_0_.

In this model, considering the movement direction of the front (towards the casting axis), a linear concentration decrease (C_l_^*^) was assumed in the liquid phase for the growth area of columnar crystals only, while with the appearance of equiaxed crystals, a faster decrease in concentration was assumed and it was described by a curvilinear correlation *C_l_*.

Generalizing the shape of crystallites in the micro scale formed from the nuclei present and/or formed near the mould wall in the form of solid and gaseous (exogenous) inclusions and on the surface of other solid (endogenous) phases depends on the value and orientation of the temperature gradient at the crystallization front, the rate of shifting the crystallization front, and the concentration gradient of the dopant in the liquid phase, before the crystallization front [42,43,44].

Modelling of microstructure formation with the use of numerical methods is already permanently present in the issues of crystallization and solidification of aluminium and iron alloys. The aim is to predict casting structures with desired local properties, and this is a basic expectation. Most often, this prediction is an indirect inference (based on the temperature gradients and the cooling rate calculated during the simulation process) or with the application of deterministic models. The selected models are presented below. They are used to predict the structure on the scale of trial castings and those made with the use of special casting technologies, e.g., casting with directional crystallization of superalloys. These are models named after the methods of solving them: Phase-Field Method, Pseudo Front Taracking and Cellular Automaton Finite Element (CAFE) [1,4,14,15,27,45,46,47]. There are two versions of the CAFE code—2D and 3D—and both codes will be applied in the paper. The advantage of 3D code over 2D depends the possibility of predicting products of practically any shape. In 2D code prognosis are possible for simple shapes: plate, axisymmetric figures introducing specific boundary conditions of heat transfer. The disadvantage of 3D codes in relation to 2D is longer CPU time.

## 3. Materials and Methods

### 3.1. Structure Prediction Using Pseudo Front Tracking (PFT) and Phase Field (PHF) Codes

Two modules of the Calcosoft simulation code from the Multiphysics family: Pseudo Front Tracking (PFT) and Phase Field (PHF) [14] were developed with the intention of recreating the formation of a dendritic structure and relating to the local modelling of physical phenomena for very small areas (less than 1 mm^2^) and for a discrete division with a very small element size (microns) [43,44,45]. The assumptions made by the authors consisted in creating a coupling of models responsible for the growth of not only the primary arms, as is the case of the Calcosoft CAFE model described in detail in the next section, but also the secondary ones. As an example cited in the literature, the growth of single equiaxed crystals or at most a group of several crystals (dendrites) in 2D geometry taken out of the context of real conditions was presented [16].

**Figure 1:**
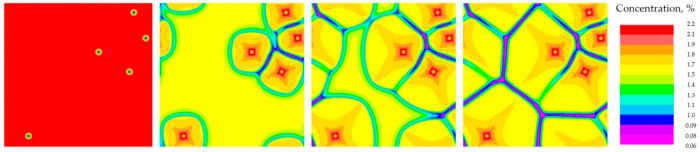
Sequence of 5-grains (simplified dendrites) growth for the simulation performed in the Calcosoft PHF (Phase Field Method) system, visible variation in solute concentration.

The following selected parameter values were used to predict the structure using the Calcosoft PHF model: number of discrete division cells in the *x* axis = 200, in the *y* axis = 200, discrete division cell size = 1.25 × 10^−6^ m, Cu concentration = 2.2% (Figure 1).

**Figure 2:**
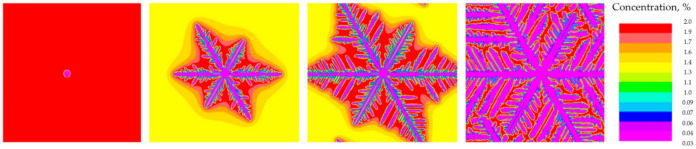
Sequence of one dendritic grain growth for the simulation performed in the Calcosoft system in the PFT (Pseudo Front Tracking) module.

**Figure 3:**
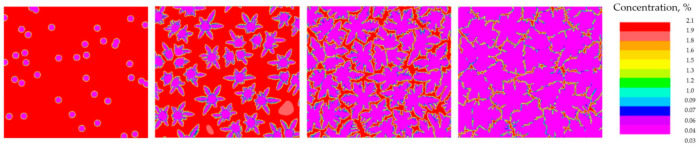
Sequence of growth of 40 dendritic grains for the simulation performed in the Calcosoft system in the PFT (Pseudo Front Tracking) module and the discretization grating with a size of 1.25 × 10^−6^ m.

Two examples of test simulations using the Calcosoft PFT module are presented below. The tests were conducted for the growth of one grain (Figure 2) and 40 grains (Figure 3). In both cases the AlCu2 alloy was used and the parameter values as in the previous case. The aim was to compare with the Calcosoft PHF model.

The above examples of structure modelling models prove that it is impossible to obtain the transfer expected by practitioners of the method of structure formation virtualization into the real object. Therefore, in this work, the Calcosoft CAFE simulation code was used in further tests. It is an example of an effective combination of macro- and micro-modelling, which enables the prediction of the structure (columnar and equiaxed pseudo-dendrites) for quite complex-shaped castings. The postulates formulated with regard to subsequent micromodels should go in this direction. Moreover, the CAFE code, unlike the others, is a part of the commercial systems of both Calcosoft and Procast. The aforementioned aspects were the basis for the validation of the CAFE code.

### 3.2. Description of the CAFE Model

The virtualization of casting processes is one of the important directions of tests on the prediction of local casting properties. This consists of identifying the property gradient and indicating areas that can bear loads exceeding the allowable stresses, which are formally averaged out of caution due to the average structure and properties of the entire casting. One way to obtain reliable information about the local properties of the casting is by modelling the crystallization process.

Modelling the phenomena responsible for crystallization, i.e., the formation of the microstructure of metal alloys during the change of their state from liquid to solid (solidification, understood in the macro scale), depending on the physical conditions of this process, was initially developed based on deterministic models describing nucleation and growth of only equiaxed (globular) crystals. These models are characterized by many simplifications and limitations, described e.g., in [22,23,48,49,50,51,52]. For this reason, models using stochastic (probabilistic) methods began to be developed.

Among the described methods and systems, the algorithms of the Calcosoft system and its CAFE module method, which is also part of one of the leading simulation systems for casting technology—ProCAST, effectively function as an advanced tool that formally enables the implementation of structure prediction in the above-mentioned way. Therefore, in this work, the validation of this simulation code was undertaken.

#### 3.2.1. Crystal Nucleation

**Figure 4:**
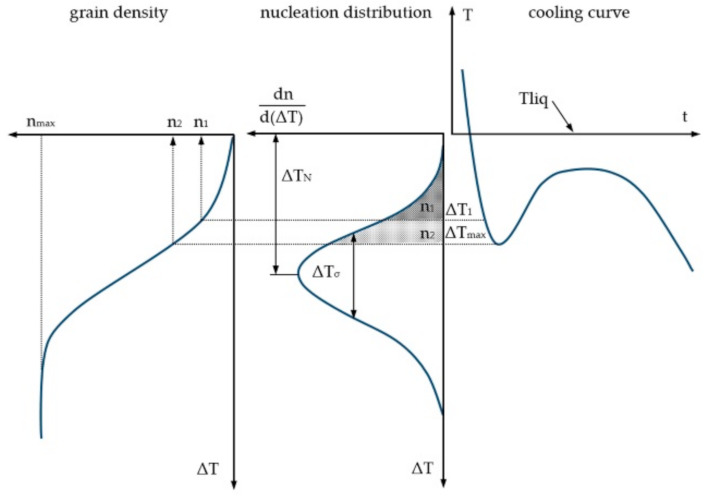
Distribution of nucleation sites and nuclei density on the mould surface n_s_ and in the bulk of liquid n_v_.

Heterogeneous nucleation, as definitely dominant in technical alloys, occurs both in the bulk of the liquid and in the liquid region at the contact surface with the mould. In the discussed model, it is described by two views derived from the basics of statistics and indicates the nucleation sites that are activated when undercooling occurs. The authors [23,48,49,50,52] proposed here the use of the normal Gaussian distribution (Figure 4) in such a way that the rate of grain formation with undercooled ΔT in the bulk of liquid *dn_v_*/*d*(Δ*T*) [m^−3^K^−1^] or on the surface of the mould *dn_s_*/*d*(Δ*T*) [m^−2^K^−1^] is calculated from Equation (1):

(1) $$\frac{dn_{v\left(s\right)}}{d\left(\Delta T\right)}=\frac{n_{max}}{\Delta T_{\sigma }\cdot \sqrt{2\pi }}exp\left[\frac{1}{2}{\left(\frac{\Delta T-\Delta T_{m-\left(i\right)}}{\Delta T_{\sigma }}\right)}^{2}\right]$$

and the distribution function of this distribution, given by the following correlation, allows to calculate the actual number of active nuclei (2):

(2) $$n_{v\left(s\right)}\left(\Delta T\right)=\int_{0}^{\Delta T}{\left[\frac{dn}{d\left(\Delta T\right)}\right]}_{v\left(s\right)}\cdot d\left(\Delta T\right)$$

where Δ*T*—local undercooling calculated using the Fourier-Kirchhoff model, Δ*T_m_*_−(*i*)_—average undercooling value corresponding to the maximum nucleation intensity, *i* = *s* (surface nucleation) or *i* = *v* (bulk nucleation), an empirical characteristic parameter of the Gaussian distribution, Δ*T_σ_*—standard deviation of undercooling, an empirical characteristic parameter of the Gaussian distribution, *n_max_*—theoretical maximum nuclei density which can be reached when all possible nucleation sites are activated during cooling, an empirical characteristic parameter of the Gaussian distribution (Δ*T_m_*_−(*i*)_, Δ*T_σ_* and *n_max_* for a given alloy are determined experimentally, taking into account the knowledge about the sensitivity of Gaussian distribution to these parameters and quantitative metallography — size and homogeneity of the grains after primary crystallization, determined a posteriori on the basis of metallographic tests), *dn_v_*—first derivative of grains number and *d*(∆*T*)—first derivative of undercooling

The above remark is a clarification of the information provided by the authors of this concept [23,48,49,53]. Modelling heterogeneous nucleation using the CA procedure is relatively simple. Each nucleating cell is assigned a critical nucleation temperature (temperature below the liquidus equilibrium temperature). For liquids deep inside the casting (for CA cells that are not adjacent to the surface of the casting), undercooling is randomly generated according to the Gaussian normal distribution and is assigned to randomly selected cells.

As mentioned above, the parameters of heterogeneous nucleation for handling the Gaussian distribution (grain density, average temperature and standard deviation) must be analysed on the basis of the experimental results obtained from the trial casting test. Corresponding undercooling ΔT_υ_^nuc^ = T_L_ − T_υ_^nuc^ (where: T_L_—liquidus temperature, T_υ_^nuc^ critical nucleation temperature) of predetermined nucleating cells is recorded. Two different Gaussian distributions are in use for the same alloy (for nucleation at the cast-mould or cast-environment surface and for nucleation in the bulk of alloy). If a cell is selected several times, then only one, lesser nucleation undercooling is used. If, during the time step, local undercooling ΔTtυ of the given nucleating υ cell which was still liquid in the preceding time step will exceed the previously assigned nucleation undercooling ΔT_υ_^nuc^, then a new grain will be created. The state index of this cell, which has a value of zero when it is in a liquid state, in this case takes a positive integer value, randomly selected from the fixed orientation class numbers [23,46].

#### 3.2.2. Crystal Growth

**Figure 5:**
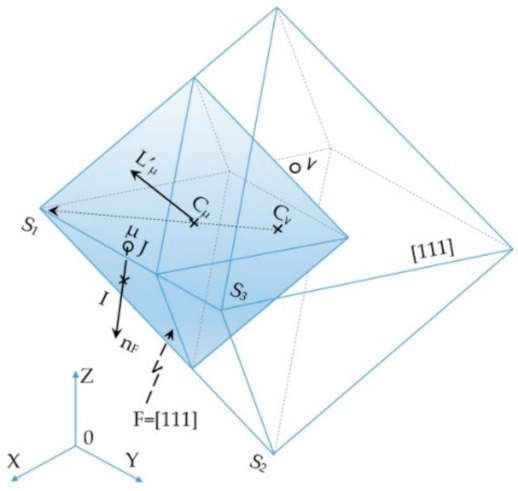
Diagram of cell growth in the CAFE 3D model.

The main diagonals of the octahedron correspond to the crystallographic orientations <100>, along which tips of metal dendrite with a regular centred wall grow in a preferred way. Their increase is calculated by integrating over time the growth equation of dendrite tips described by Kurz formula [23,48]. Therefore, in the time step *δt* used to integrate the growth rate of the dendrite tips, the increase in the length of the diagonals of the octahedron associated with the v cell (Figure 5) Δ*R_v_* is described by Equation (3):

(3) $$\Delta R_{v}=v\left(\Delta T_{v}\right)\;\cdot \delta t$$

where: ν(Δ*T_ν_*) = *a*_2_·Δ*T*^2^ + *a*_3_·Δ*T*^3^ is the dendrite tip growth rate calculated for a given undercooling Δ*T_ν_* in the cell *ν*.

### 3.3. Methodology of Experimental and Simulation Tests

#### 3.3.1. Assumption and Methodology of Experimental Tests

The cylindrical shape of the casting was adopted for the tests due to its axial symmetry and the resulting facilitations in the formulation of the thermal model and in the description of the correlation of the crystal structure parameters with the thermal parameters of the cast-mould system. The experimental studies were conducted on AlSi7Mg alloy castings. with the chemical composition presented in Table 1 with a diameter of 70 mm and height of 220 mm, made in three types of moulds: homogeneous moulding sand (Q-Q), a moulding sand (Q) with a copper chill (Ch) located on the bottom side of the cylindrical casting, and in the mould of insulating mass (HI) with an analogously placed copper chill (Ch).

The parameters of the resulting structure will be described (for solid solution dendrites α and especially the location of the columnar-to-equiaxed transition zone—CET). The number of equiaxed crystals per unit area was also estimated.

A method of instrumentation of the mould (distribution of thermocouples) was developed, which allowed for the quantitative identification of thermal conditions, and then enabled the validation of the model used in the simulation of the crystallization process. The crystallization model used (Calcosoft, CAFE module) incorporates only the growth of solid solution dendrites α (as CAFE does not incorporate the crystallization of the eutectic phase and the intermetallic phases). Possible estimation of the presence of the eutectic phase can only concern the zones between the simplified blocks of dendritic crystals. However, this is not a direct result of the virtualization of the crystallization process of all phases of this alloy. The intensity of heat abstraction from the casting to the mould and/or chill, and thus the casting solidification process, was defined by the local solidification times of the α phase determined on the basis of the recorded cooling curves.

#### 3.3.2. Assumption and Methodology of Simulation Tests

As mentioned above, the Calcosoft system with the CAFE-3D module was used in the simulation studies to predict the pseudo-dendritic structure. The simulation tests concerned a cylinder-shaped casting with a diameter (ϕ) of 70 mm. Firstly, the energy validation of the real and virtual solidification time compliance was performed by interpreting the cooling curves on the basis of their first derivatives. The validation concerned only the solidification of the α phase, as the Calcosoft CAFE model considers only the growth of this phase and formally assumes that it fills the entire space of the structure, including the eutectic phase that actually surrounds it.

Therefore, the pseudo-dendritic virtual structure (in the form of compact pseudo-grains) should be treated as the α phase dendrite filled in the central part of the pseudo-grain, and its environment is the eutectic phase invisible on the virtual structure. Estimation of the proportions of both phases in a pseudo-grain can be performed using the classical lever rule (assuming thermodynamic equilibrium).

In the next stage, tests related to the sensitivity of the CAFE model was conducted, i.e., by checking the impact of changing the values of individual parameters available in the CAFE model on the position of columnar-to-equiaxed transition zone and the grain size (pseudo-dendrites). The ranges of parameter values for this procedure were selected. Only one parameter value was variated at once in these studies.

Validation studies related to structure prediction consisted in the analysis of the compliance of the CET zone location and grain size. The problem of the influence of the variability of the model parameters on the orientation of pseudo-dendrites in the casting space was also analysed. The values of the orientation angles of the columnar grain blocks from the calculation are marked by colour differentiation.

#### 3.3.3. Experimental and Simulation Validation

**Figure 6:**
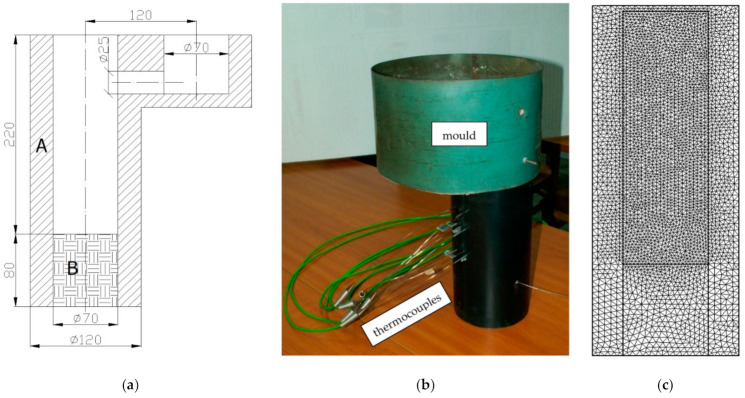
Casting mould: (**a**) mould diagram (A—moulding sand (Q) or insulating mass mould (HI) material B—moulding sand (Q) or chill (Ch)), (**b**) mould view, (**c**) virtual mould with FEM mesh.

In the simulation calculations, variants of selected values of thermophysical parameters and parameters related to the model of microstructure formation were used. Table 1 presents their values for the tests of the casting solidifying in the following moulds: homogeneous moulding sand (Q-Q), of moulding sand with a chill (Q-Ch), of an insulating mass with a chill (HI-Ch). The diagram of the mould, its real view and the axial section of the virtual mould with the visible FEM discrete mesh are shown in Figure 6.

It should be added that the introduction in the CAFE-3D module of modelling parameters for the casting crystallization including the segregation of the solute alloy component (Si) is an important distinguishing feature of the model coupling. It is based on the fact that the heat generation of crystallization is related to the liquidus temperature updated at each time step during the simulation, in a function of the local concentration of the solute. The concentration variation is made according to the Scheil model (Equation (4)). It assumes that the solute does not diffuse in the solid phase, but that it is fully distributed in the liquid phase:

(4) $$C_{S}=kC_{0}{\left(1-f_{S}\right)}^{k-1}$$

where *k*—partition coefficient (*k* = *C_S_*/*C_L_*), *C_S_*—solute concentration in the solid phase, *C_L_*—solute concentration in the liquid phase, *C*_0_—initial solute concentration and *f_S_*—fraction of solid.

The analysis and selection of thermophysical coefficients and the values of the parameters necessary for empirical correlations (nucleation and growth) required for the simulation, leading to good compliance of the virtually predicted structure with the structure from the experiment, are easier in terms of conscious selection of the abovementioned parameters when their impact on the predicted structure is known (model sensitivity). Table 2 lists the ranges of parameter values that were determined as a result of simulation tests. They are a result of many series of simulations under various thermal conditions in the cast-mould system.

The thermophysical parameters of the mould that have a decisive influence on the intensity of heat receive from the liquid phase are λ, c and ρ of the moulding sand and the heat transfer coefficient—α on the casting-mould interface (casting—chill).

The simulation tests of the cooling curves were conducted by varying the λ values of the moulding masses and α cast-mould (casting-chill), and the remaining values of the thermophysical parameters of the thermal model were assumed to be of constant value:

- heat capacity of AlSi alloy of the solid phase—cρ_Al-sol_ = 2700 kJ/(m^3^K), of the liquid phase cρ_Al-liq_ = 3483 kJ/(m^3^K);
- thermal conductivity of alloy of the solid phase λ_Al-sol_ = 130 W/(mK), of the liquid phase λ_Al-liq_ = 90 W/(mK);
- latent heat of solidification L = 1.131 × 10^9^ J/m^3^; heat capacity of the moulding sand—cρ_Q_ = 1500 kJ/(m^3^K);
- heat capacity of the insulating mass—cρ_HI_ = 587 kJ/(m^3^K); heat capacity of the copper chill—cρ_Ch-Cu_ = 3300 kJ/(m^3^K) and thermal conductivity of the chill λ_Ch-Cu_ = 390 W/(mK), and the following parameters necessary to enter into the Scheil model (Equation (4)) and the solid phase increase generation model:
- pure aluminium (Al) melting point—660 °C,
- eutectic temperature T_eut_ = 572 °C,
- initial solute concentration (Si) c_0_ = 7% and the angle of the liquidus line slope m = −6.85°.

## 4. Results

The results of the tests on the influence of the parameter values in the CAFE model on CET and crystal size d_av_ were analysed on the basis of virtual structures for solidifying castings in the tested three configurations of moulds: Q-Q, Q-Ch and HI-Ch.

Analysis of the impact of the CAFE—3D model parameter values on CET and the average crystal size (pseudo-grains) *d_av_* was determined from the Equation (5):

(5) $$d_{av}=\frac{4n_{L}}{\pi \cdot n_{S}}$$

where *n_L_*—number of intersections of grains per unit length and *n_S_*—number of grains per unit area, which has been described separately for each material combination of the mould.

For each of the three variants of the mould configurations, the virtual structure best fitted to the experimental one is presented.

### 4.1. Validation of the CAFE-3D Model on the Basis of the Casting Solidifying in the Q-Q Mould

The analysed parameters of the virtual structure of the casting made in the Q-Q mould are subject to the following influences:

- in the thermal model—an increase in the thermal conductivity coefficient λ (mould) from 0.5 to 2.0 W/(mK) causes an increase in the CET zone in the casting layer near the mould surface by approx. 10 mm, with no significant influence of λ on the crystal size d_av_ by approx. 0.1 mm,
- in the thermal model—an increase in the heat transfer coefficient (cast-mould) α_cast-Q_ from 100 to a high value of 10,000 W/(m^2^K) does not significantly affect the position of CET (imperceptible variability in the near-surface layer of the casting) and d_av_, which is the result of the dominant effect of thermal resistance resulting from the presence of the moulding sand layer,
- in the nucleation model—undercooling in the bulk of the liquid phase ΔT_m-v_ in the range from 2 to 5 °C has no effect on CET and slightly increases the d_av_ by approx. 0.13 mm, only above this value of undercooling a longer CET zone begins to form, reaching a value variation of approx. 35 mm applying 10 °C and an increase of the grain size d av by approx. 2 mm,
- in the nucleation model—increase in nuclei density in the bulk of the liquid phase n_v_ from 1 × 10^5^ to 1 × 10^9^, m^−3^ has a large impact on reducing CET by approx. 23 mm and d_av_ 4.7 mm,
- in the growth model—increase in the value of the kinetic coefficient a_3_ in the pseudo-dendrite growth rate equation from 1.5 × 10^−8^ to 1.5 × 10^−5^ ms^−1^K^−3^ causes a large increase in CET by approx. 40 mm and d_av_ of approx. 3.46 mm.

**Figure 7:**
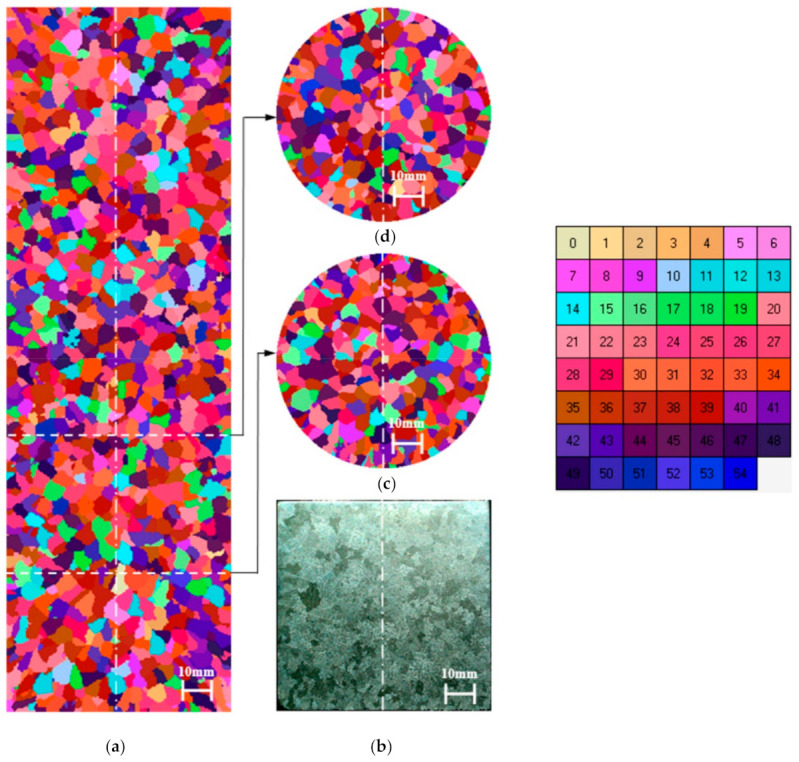
Setting of structures for casting solidifying in a homogenous mould made of moulding sand (Q-Q): (**a**) virtual structure (longitudinal section), (**b**) real structure (longitudinal section), (**c**) virtual structure in height of 44 mm from the base of the cylinder (cross-section), (**d**) virtual structure in height of 88 mm from the base of the cylinder (cross-section). The CET zone does not occur due to the lack of forced heat flux. The samples were etched: 5 mL HF + 20 mL HCl + 20 mL HNO_3_ + 55 mL of H_2_O. Parameters used for the simulation: λ_Q_ = 0.5 W/(mK), α_odl-m_ = 10,000 W/(m^2^K), ΔT_m-s_ = 5 K, n_v_ = 1 × 10^7^ m^−2^, n_s_ = 1 × 10^5^ m^−2^, ΔT_m-v_ = 2 K, a_3_ = 1.49 × 10^−7^ m s^−1^ K^−3^.

**Figure 8:**
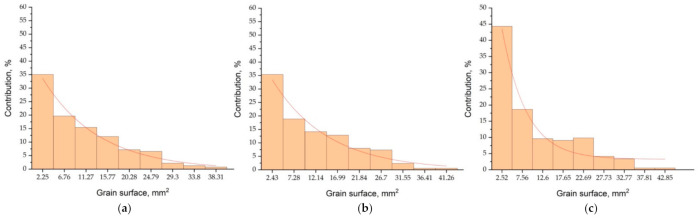
Surface histograms according to division into classes of pseudo-grains of the virtual structure (Figure 7) on the cross-section at distances from the base of the cylinder: (**a**) 44 mm, (**b**) 88 mm, (**c**) 116 mm.

Comparison of the virtual structure with the real one (Figure 7), the results of the average grain size (Table 3), and the distribution of the grain surface share (Figure 8), indicate a satisfactory match in the near-surface layer and the rest of the casting.

### 4.2. Validation of the CAFE-3D Model on the Basis of the Casting Solidifying in the Q-Ch Mould

**Figure 9:**
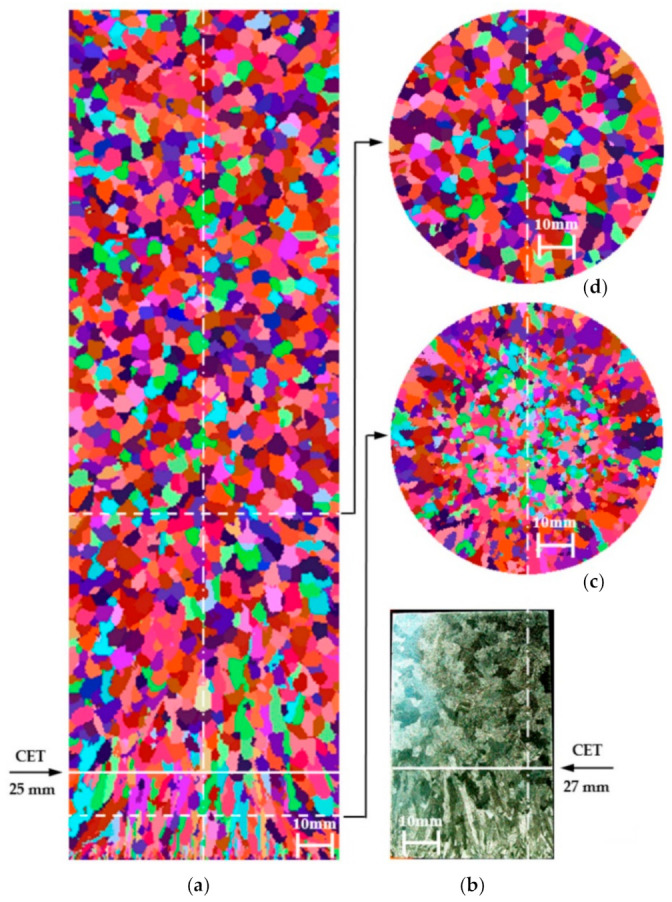
Setting of structures for casting solidifying in a uniform mould made of sand mass with a chill (Q-Ch): (**a**) virtual structure (longitudinal section), (**b**) real structure (longitudinal section), (**c**) virtual structure in height of 10 mm from the base of the cylinder (transverse cross-section), (**d**) virtual structure in height of 88 mm from the base of the cylinder (transverse cross-section). The samples were etched with 5 mL HF + 20 mL HCl + 20 mL HNO_3_ + 55 mL of H_2_O. Parameters used for the simulation: λ_Q_ = 1.0 W/(mK), α_odl-m_ = 10,000 W/(m^2^K), α_odl-Q_ = 4000 W/(m^2^K), ΔT_m-s_ = 5 K, n_s_ = 1 × 10^6^ m^−2^, ΔT_m-s-Ch-Q_ = 15 K, n_s-Ch_ = 5 × 10^6^ m^−2^, ΔT_m-v_ = 2 K, n_v_ = 2 × 10^7^ m^−2^, a_3_ = 2 × 10^−10^ m s^−1^ K^−3^.

Validation studies of the model with regard to the structure (in macro terms) of the casting solidifying in the mould marked Q-Ch were carried out using the parameter values in the ranges given in Table 2 for the simulation. These values were selected successively based on the CET value analysis of virtual structures obtained from subsequent simulations. The virtual structure best fitted to the real structure is shown in Figure 9.

**Figure 10:**
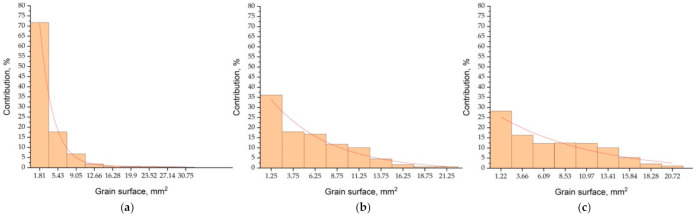
Surface histograms according to division into classes of pseudo-grains of the virtual structure (Figure 9) on the transverse cross-section at distances from the base of the cylinder: (**a**) 10 mm, (**b**) 44 mm, (**c**) 88 mm.

The specification made on the basis of the grain surface distribution (Table 4 and Figure 10) of the average size of the columnar crystals on the cross-section (z = 10 mm) and equiaxed (z = 88 mm) of the virtual structure equal d_av_ = 2.91 mm and 4.28 mm shows a very good match to the real areas of the etched structure, amounting to d_av_ = 2.95 mm and 4.31 mm, respectively.

The virtual structure on the longitudinal section shown in Figure 9a correctly predicts the extent of the CET zone (27 mm), the angle of their inclination in relation to the vertical axis of the casting (in the lower, central zone of the casting—the direction of heat flux preferred by the presence of a chill). In a virtual structure, in the lower central part of the casting with a radius of approx. 17 mm, the γ angle is 4–14°, and in the outer layer above the radius of 17 (up to 35 mm) the γ angle is 7–42°, respectively. Accordingly, the real structure (in the central part of the casting with a radius of approx. 17 mm is characterized by the γ angle of 2–25°, and in the layer above the radius of 17 (up to 35 mm), the γ angle is 4–33°. The virtual structure in height of z = 10 mm from the casting base contains a large number of columnar crystals, i.e., 1291 and this number is well approximated to the experimental one of 1221 (a difference of approx. 5.4%).

The reduction of the γ angle of inclination of the columnar crystals in relation to the vertical axis of the casting, taking into account the degree of their mutual orientation, should be carried out by influencing the virtual structure by increasing the value of α_cast-Ch_ over 3000 W/m^2^K and decreasing the value of a_3_ under 5 × 10^−10^ m s^−1^ K^−3^. Increasing the value of α_cast-Ch_ always increases the CET value. The reduction of the CET value can be done by increasing the number of grains in the bulk of the liquid phase n_v_.

### 4.3. Validation of the CAFE-3D Model on the Basis of the Casting Solidifying in the HI-Ch Mould

**Figure 11:**
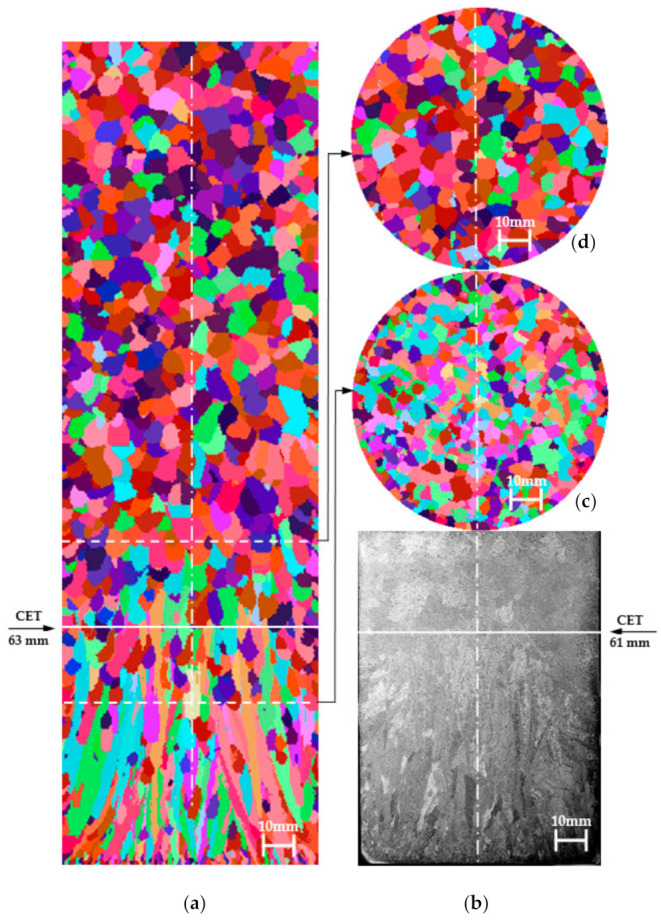
Setting of structures for casting solidifying in a uniform mould made of insulating mass with a chill (HI-Ch): (**a**) virtual structure (longitudinal section), (**b**) real structure (longitudinal section), (**c**) virtual structure in height of 44 mm from the base of the cylinder (transverse cross-section), (**d**) virtual structure in height of 88 mm from the base of the cylinder (transverse cross-section). The samples were etched: 5 mL HF + 20 mL HCl + 20 mL HNO_3_ + 55 mL of H_2_O. Parameters used for the simulation: λ_HI_ = 0.5 W/(mK), α_odl-m_ = 10,000 W/(m^2^K), α_odl__-Ch_ = 2500 W/(m^2^K), ΔT_m-s_ = 5 K, n_v_ = 8 × 10^6^ m^−2^, n_s_ = 1 × 10^6^ m^−2^, ΔT_m-v_ = 2 K, a_3_ = 5 × 10^−9^ m s^−1^ K^−3^.

Validation studies of the model of creating the casting structure solidifying in the HI-Ch mould were carried out on the basis of the analysis of the parameter values adopted for simulation in the ranges given in Table 2. These values were selected successively based on the CET value analysis of virtual structures obtained from subsequent simulations. The virtual structure best fitted to the real structure is shown in Figure 11, the values of the virtual and experimental structure parameters are presented in Table 5.

The obtained virtual structures and their values: CET (Figure 11), size d_av_ (Table 5) indicate that parameters with values n_v_ ≤ 1 × 10^7^ m^−3^ and a_3_ ≤ 1 × 10^−8^ m·s^−1^·K^−3^ enable us to obtain a satisfactory approximation of the CET and d_av_ of the real structure, visible on the etched sample. The average size of equiaxed crystals on the transverse cross-section (z = 10mm) of the virtual structure (d_av_ = 2.9 mm) is very well matched to the real areas of the etched structure (d_av_ = 3.0 mm). The virtual structure at height of z = 44 mm from the casting base contains 689 columnar crystals and this number is well approximated to the experimental one of 658 (difference of approx. 4.5%). The greatest distance of the CET zone of the virtual structure is approx. 63 mm, while in the real casting it is approx. 61 mm (central part of the casting). The angle of inclination of the bar crystals measured in regard to the vertical axis of the casting at a distance (radius) of 15 mm from the axis, i.e., the central part of the casting, is 0–10° for both the experimental and virtual structure. However, in the remaining area for the experimental structure, the angle γ is 16–48°with respect to the virtual structure, where γ is 12–45°. The greatest inclination angles of the crystals are observed from the side of contact with the chill at the edge of the casting. Therefore, also a good approximation of the virtual structure to the real one was obtained due to the angle of the crystals.

The analysis of the test results shows that a relatively good approximation of the virtual and real structure of the casting solidifying in the HI-Ch mould can be obtained by using the following parameter values for the CAFE-3D model:

- parameters assumed to be of constant value: α_cast-m_ = 10,000 W/(m^2^ K), ΔT_m-s_ = 5 K, ΔT_m-s-Ch-HI_ = 10 K, ΔT_m-v_ = 2 K, σ_ΔTs-HI_ = 0.4 K, σ_ΔTs-Ch_ = 0.4 K, σ_ΔTv_ = 0.4 K,
- parameters with values assumed to be variable in the range: λ_Q_ = 0.3–0.5 W/(m K), α_cast-Ch_ = 1500–2500 W/m^2^K, n_s_ = (1–5)10^5^ m^−2^, n_s-Ch_ = (8–10)10^5^ m^−2^, n_v_ = (8–10)10^6^ m^−3^, a_3_ = (1–3) 10^−9^ m s^−1^ K^−3^.

The reduction of the γ angle of the inclination of the columnar pseudo-dendrites and their degree of mutual orientation should be adjusted by increasing the value of α_cast-Ch_ over 2500 W/m^2^K and decreasing the value of a_3_ under 3 × 10^−9^ m s^−1^ K^−3^.

### 4.4. Example of Predicting Microstructure under Conditions of Extreme Heat Transfer

**Figure 12:**
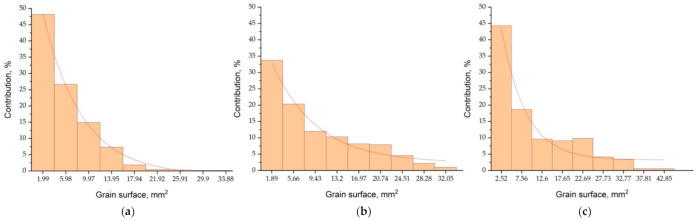
Surface histograms according to division into classes of pseudo-grains of the virtual structure (Figure 11) on the transverse cross-section at the distances Z: (**a**) 44 mm, (**b**) 88 mm, (**c**) 116 mm.

Moreover, an attempt was made to predict the microstructure resulting from the application of a diode laser, the beam of which influences the surface of the AlSi7Mg alloy sample made in the die-casting mould (Figure 12). This method is used to improve the tribological properties by significantly crumbling the grain in the surface layer and alloying it [54,55,56,57]. The laser heat treatment process was carried out using a TRUMPF TruDiode 3006 diode laser (TRUMPF, Ditzingen, Germany) with a maximum power of 3 kW. To manipulate the location of the laser beam a KR16-2 robot (KUKA, Augsburg, Germany) was used. Beam diameter d = 2.18 mm, power 1800 W, velocity 5.5 m/s, energy per surface 150.1 J/mm^2^.

In these tests, the Calcosoft–CAFE simulation code was used, which is the prototype of the 3D version. An important problem encountered in this process was the method of determining the values of the parameters (the thermal model and the model responsible for structure prediction) under extreme heat extraction conditions during laser remelting of the casting surface and its subsequent rapid crystallization. Due to the very high heating/cooling rates and a relatively small remelting area, it was necessary to use a very small FEM mesh size of 0.02 mm (in the conditions of classic casting 1 mm and more) and a cellular automaton grid from 1 to 5 μm (in the conditions of classic casting 100 μm) and to drastically reduce the time step to 1 × 10^−8^ s (the case with a copper chill is approx. from 0.5 to 1 s).

Moreover, it was necessary in the database defined for the CAFE module to significantly increase the value of the heat transfer coefficient (melted drop—non-melted material system) up to the value of 1 × 10^8^ W/m^2^K (cast-chill from 2500 to 4000 W/m^2^K). Whereas, the value of the empirical coefficient entering the correlation on nuclei density at the interface liquid phase—non-melted material should be assumed as n_casting-mould_ = 1 × 11 m^−2^ (case with chill n_casting-mould_ = 1 × 10^5^ m^−2^).

**Figure 13:**
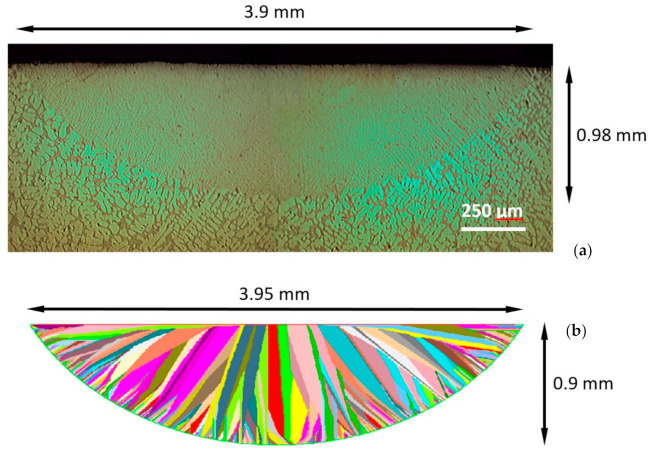
Result of experimental and simulation validation in the Calcosoft CAFE 2D code: (**a**) real microstructure (Nikon ECLIPSE MA200 microscope), (**b**) virtual microstructure.

The simulation results were consistent with the experiment. In Figure 13a, the actual structure is shown with a visible non-melted material and melted zone. There is no visible transition zone from columnar to equiaxed (CET) crystals between the two zones as was in the case of the variants described in the previous section. In places bordering with the non-melted zone, the formation and growth of fragmented columnar crystals can be observed without a gradual reduction of the structure grains. Columnar crystals are formed in the non-melted material structure as a result of the forced large heat flux by removing the heat of the casted zone through the non-melted zone. On the virtual structure (Figure 13b), the formation and growth direction of columnar crystals formed at the interface with the non-melted material zone can be observed, due to the forced large temperature gradient from the contact zone with the non-melted zone, spreading towards the contact of the melted layer with the ambient. Both in the real and virtual structure, the formation of equiaxed crystals was not observed, due to specific thermal conditions, i.e., forced large heat flux.

In the next stage, microscopic examination (ECLIPSE MA200 microscope, Nikon, Tokyo, Japan) of the microstructure refinement was performed. The parameter determining the refinement of the structure is the dendrite arm spacing (DAS). This parameter was obtained on the basis of arithmetic average measurements of dendrite arm distances in the non-melted material and melted zones. Therefore, in the non-melted zone, the average values were 41 µm, while in the melted zone 4.5 µm. Consequently, the refinement of the solid α solution was performed nine times.

The same sample was subjected to microscopic examination using a microhardness tester to determine the Vickers micro-hardness (VMH 002VD microscope type microharness tester, Walter UHL Technische, Aßlar, Germany). The applied force was HV 0.025 of 0.2452 N. The test was performed in accordance with EN ISO 6507-1:2005. A Vickers microhardness of 60–71 HV 0.025 (66 HV 0.025 on average) was obtained in the non-melted zone. In the melted zone, the microhardness significantly improved and ranged from 85–92 HV 0.025 (88 HV 0.025 on average). Therefore, the refinement of the structure caused by the high cooling rates increased the relative difference between the microhardness obtained in the melted and non-melted zone by 25%. This has an obvious effect on the improvement of the tribological properties.

## 5. Discussion

A collective summary of the test results related to the validation of the Calcosoft CAFE 3D model is presented in Table 5. The Calcosoft database is very poor, and additionally the publications of the system creators do not provide a wide range of parameter values. Therefore, first of all it was necessary to test the sensitivity of the CAFE model to variations in the values of individual parameters, and then to adjust the selected cases of the virtual structure to the real one (Q-Q, Q-Ch, HI-Ch), which enabled the determination of their reasonable (real) limits of applicability (application). It is therefore a very extensive attempt to expand the database of the above-mentioned simulation system. Table 6 shows the adopted ranges of the CAFE model parameter values, indicating the values of the parameters for which the best matching of virtual structures to the real ones was obtained for individual material configurations of moulds, taking into account the location of the CET zone, as well as the average grain size—d_av_. The recommended specified ranges of variations of the values of the CAFE model parameters for the comparative analysis of the deviation angle from the direction forced by the chill influence of directed pseudo-crystallites of the virtual structure and dendrites of the real structure are also given.

The analysis of the results of the validation tests shows that in the prediction of the structure using the Calcosoft CAFE-3D model of the solidifying casting in the thermal conditions of the mould (Q-Q, Q-Ch, HI-Ch) assumed in the operation, it results that the influence of individual parameters used in these coupled models the simulation result is varied. This impact was estimated using the notation in Table 5—! for little impact, !!—for medium and !!! for high one. This applies to both groups of parameters, i.e., those occurring in the thermal model and the model related to the phenomena of nucleation and structure growth. If there is a need to correct the match of the virtual structure to the real one, it should be made by adjusting the value of the nuclei density parameter in the bulk of casting—n_v_, and when the length of the CET zone is reduced too low, then it should be increased, first of all by increasing the kinetic coefficient—a_3_ and then by increasing the value of the heat transfer coefficient at the cast-chill interface—α_cast-Ch_.

The reduction of the γ angle of the columnar crystals in relation to the vertical axis of the casting (the direction of the main component of the flux preferred by the location of the chill), taking into account the degree of their mutual orientation, should be analysed by affecting the virtual structure by increasing the value of the heat transfer coefficient at the casting and chill interface—α_cast-Ch_ and by reducing the value of the kinetic coefficient—a_3_. Increasing the value of α_cast-Ch_,as mentioned above, will always increase the value of the CET zone. Whereas, the reduction of the CET zone value in this case can be achieved by increasing the density of the grains in the bulk of the liquid phase—n_v_. These activities specifying the validation of the structure formation model go beyond the area of two basic predicting parameters (virtual structure), which are the CET zone position value and the average grain size d_av_ (see Table 7). This requires a good orientation of the CAFE system user in the sensitivity issues of this coupled model, which is shown in Table 1.

## 6. Conclusions

The influence of individual parameters on the simulation result was determined. Therefore, the following conclusions will be of a general nature and will provide guidelines for further work.

The conducted analysis of the state of knowledge about the basics of crystallization of metals and alloys and their use to predict the structure in castings by means of coupled modelling, a review of the methods and simulation systems used, and most of all the experimental and simulation studies carried out on the CAFE system validation enable the following summary:

(1) From the phenomena accompanying the creation of a structure in a real casting, coupled in a way resulting from the nature of the process, the most important of them were selected, namely the heat flux in the cast-mould system (thermal conductivity of the moulding sand, heat transfer coefficient on the cast-mould and cast-chill interface) and nucleation and crystal growth phenomena (undercooling at the mould surface, number of nuclei at the mould surface, undercooling at the chill surface, number of nuclei at the chill surface, undercooling in the bulk of casting, number of grains in the bulk of casting, kinetic growth coefficient a_3_).
(2) The developed methodology and designed stands for conducting experimental and simulation validation studies met the assumptions of a modern, special and comprehensive validation procedure, going beyond the validation of thermal phenomena. It was shown that it was necessary to adequately “isolate” the mentioned modelled coupled phenomena in terms of interpretation.
(3) A set of model parameter values was developed for castings with a diameter of ϕ70 mm, which enabled the most accurate prediction of the AlSi7Mg alloy structure in the chill impact zone and outside its zone (in this dimensional area of castings cooled intensively from the bottom side).
(4) Also, the algorithms were included that enable to predict the structure defined by the following parameters:
  - degree of refinement (grain size—d_av_) with the interpretation of the restriction d_av_ to the hypoeutectic phase,
  - location of the columnar-to-equiaxed transition zone—CET
  - angle of the crystals in relation to the vertical axis of the casting—γ.
(5) The CAFE model requires a special approach in the selection of parameter values under conditions of intense heat transfer, such as the case of rapid remelting and rapid solidification of small volumes of the alloy. It is necessary to enrich the database of materials and the database of boundary conditions with appropriate values depending on the intensity of heat transfer. In order to better match the thermal conditions in such cases, and to better identify variations in temperature fields with time, it is worth using the IR thermography method [58].
(6) The above conclusion in some approximation (not so extreme) also applies to variants using chills in classic heat removal conditions.
(7) The next step in modelling the microstructure should be to achieve dendritic structures in three-dimensional modelling for entire castings. Moreover, it includes the occurrence of eutectics in the interdendritic spaces. As well as the transfer of the obtained structure parameters (distances between the arms of the dendrites—DAS, i.e., the degree of their refinement, the size of the eutectic particles) on the mechanical properties (tensile strength, accepted yield point, elongation, hardness). It is now necessary to develop new empirical models, which until now were mainly related to the cooling rate of the alloy.

## Figures and Tables

**Table 1:** Chemical composition of the alloy to be tested (AlSi7Mg).

Element	Si	Mg	Cu	Fe	Ti	Mn	Zn	Al
Concentration %	6.86	0.3	0.01	0.1	0.01	0.01	0.01	bal.

**Table 2:** Ranges of parameter values used for simulation.

Parameter	Name	Value	Unit
**Changing-value Parameters**			
homogenous silica sanding mould			
λ_Q_	heat conduction	0.5–1.5	W/mK
α_cast-mould_	heat transfer coefficient (HTC)	500–20,000	W/m^2^K
ΔT_m-s-Q_	undercooling on the mould surface	2–5	K
ΔT_m-v-Q_	undercooling in the bulk of liquid	2–10	K
n_s-Q_	nuclei number on the mould surface	1 × 10^4^–1 × 10^8^	1/m^2^
n_v_	nuclei number in the bulk of liquid	1 × 10^5^–1 × 10^10^	1/m^3^
a_3_	kinetic coefficient	1.49 × 10^−8^–1.49 × 10^−5^	ms^−1^K^−3^
double-material silica sanding mould with chill			
λ_Q_	heat conduction	0.5–1.5	W/mK
α_cast-chill_	heat transfer coefficient (HTC)	100–800	W/m^2^K
ΔT_m-v-Q_	undercooling in the bulk of liquid	1.5–5	K
n_v-Q_	nuclei number in the bulk of liquid	1 × 10^6^–1 × 10^9^	1/m^3^
a_3_	kinetic coefficient	2 × 10^−7^–2 × 10^−5^	ms^−1^K^−3^
double-material silica sanding mould with chill			
λ_HI_	heat conduction	0.3–1.5	W/mK
α_cast-chill_	heat transfer coefficient (HTC)	50–1000	W/m^2^K
ΔT_m-s-HI_	undercooling on the mould surface	2.5–10	K
ΔT_m-s-ch-HI_	undercooling on the chill surface	5–20	K
ΔT_m-v-HI_	undercooling in the bulk of liquid	0.5–8	K
n_s-HI_	nuclei number on the mould surface	1 × 10^5^–1 × 10^8^	1/m^2^
n_s-CH_	nuclei number on the chill surface	1 × 10^5^–1 × 10^8^	1/m^2^
n_v-HI_	nuclei number in the bulk of liquid	1 × 10^7^–1 × 10^10^	1/m^3^
a_3_	kinetic coefficient	6.5 × 10^−8^–6.5 × 10^−5^	ms^−1^K^−3^
**Fixed-value parameters**			
α_cast-mould_	heat transfer coefficient on the casting-mould interface	10,000	W/m^2^K
α_mould-ambient._	heat transfer coefficient on the mould-ambient interface	20	W/m^2^K
C_pQ_	heat capacity of mulding sand	1500	kJ/m^3^K
C_pHI_	heat capacity of isolating mulding material	587	kJ/m^3^K
σ_ΔTs-Q/HI_	standard deviation on the mould surface	0.4	K
σ_ΔTs-Ch_	standard deviation on the chill surface	0.4	K
σ_ΔTv_	standard deviation in the bulk of liquid	0.4	K
ΔT_m-s-Q_	undercooling on the mould surface (Q-Ch)	5	K
ΔT_m-s-ch-Q_	undercooling on the chill surface (Q-Ch)	15	K
n_s-Q_	nuclei number on the mould surface (Q-Ch)	1 × 10^6^	1/m^2^
n_s-Q_	nuclei number on the chill surface (Q-Ch)	1 × 10^7^	1/m^3^

**Table 3:** The values of the parameters of the Q-Q casting structure (Figure 7).

	Cross Section	Virtual Structure				Exp. Structure
Structure Parameters—CAFE		Z = 44 mm Crosswise	Z = 88 mm Crosswise	Z = 116 mm Crosswise	Y = 0 mm	Z = 44 mm Crosswise
Total grain number (Nb)		384	353	357	1321	356
Grains density on mould surface, 1/m^2^		99 811	91 754	92 793	85 779	92 520
Average grain size d_av_, mm		5.0	5.2	5.2	5.5	5.2

**Table 4:** The values of the structure parameters (see Figure 9) Q-Ch variant.

	Cross Section				Exp. Structure	
Structure Parameters—CAFE		Z = 10 mm Crosswise	Z = 44 mm Crosswise	Z = 88 mm Crosswise	Z = 10 mm Crosswise	Z = 88 mm Crosswise
		Columnar	Equiaxed		Columnar	Equiaxed
Total grain number (Nb)		1291	681	558	1221	512
Grains density on mould surface, 1/m^2^		335,564	177,009	145,038	315,963	133,120
Average grain size d_av_, mm		2.91	3.75	4.28	2.95	4.31

**Table 5:** The values of the structure parameters (Figure 11).

	Cross Section	Virtual Structure				Exp. Structure
Structure Parameters—CAFE		Z = 44 mm	Z = 88 mm	Z = 116 m	Y = 0 mm	Z = 44 mm
		Columnar	Equiaxed			Columnar
Total grain number (Nb)		689	444	393	1417	658
Grains density on mould surface, 1/m^2^		179 088	114 076	102 150	92 012	171 013
Average grain size d_av_, mm		4.06	4.64	5.09	5.06	4.1

**Table 6:** Summary of validation results for the casting with greater massiveness in all three moulds: Q-Q, Q-Ch and HI-Ch.

Model	Parameter	Symbol and Unit	Value or Range of the Tested Values of the Model Parameters and Influence in the Model (High—!!!, Middle—!!, Low—!)			Parameter Values for the Best Fit of the Model for CET and d_av_			Recommended Ranges of Parameter Value Changes when Varianting the Position Pseudocrystals Angle (γ) while Maintaining the CET Value	
			Q-Q	Q-Ch	HI-Ch	Q-Q	Q-Ch	HI-Ch	Q-Ch	HI-Ch
Thermal	Heat conduction of the mould	λ_Q/HI_ W/(mK)	0.5–2 (!)	0.5–1.5 (!)	0.2–1.5 (!!)	0.5	1.0	0.5	0.75–1	0.3–0.5
	HTC casting-mould	α_cast-m_ W/(m^2^K)	100 – 10,000 (!)	10,000 (!)	10,000 (!)	10,000	10,000	10,000	10,000	10,000
	HTC casting-chill	α_cast-Ch_ W/(m^2^K)	-	100–4000 (!!)	50–5000 (!!)	-	4000	2500	3000 – 4000	1500 – 2500
Nucleation and growth (CAFE)	Undercooling at mould surface	ΔT_m-s_ K	2–5 (!)	5 (!)	2.5–10 (!)	5	5	5	5	5
	Nuclei Number at mould surface	n_s_ 1/m^2^	1 × 10^4^ – 1 × 10^8^ (!!)	1 × 10^3^ – 1 × 10^7^ (!!)	1 × 10^5^ – 1 × 10^8^ (!!)	1 × 10^5^	1 × 10^5^	1 × 10^5^	(1–5)10^5^	(1–5)e5
	Undercooling at chill surface	ΔT_m-s-Ch-Q/HI_ K	-	10–15 (!)	5–20 (!)	-	15	10	10–15	10–15
	Nuclei number at chill surface	n_s-Ch_ 1/m^2^	-	5 × 10^5^ – 5 × 10^7^ (!)	1 × 10^5^ – 1 × 10^8^ (!)	-	5 × 10^6^	8 × 10^5^	(1÷–5)10^7^	(8–10)10^5^
	Undercooling at the bulk of liquid	ΔT_m-v_ K	1.5–10 (!!!)	1.5–5 (!!!)	0.5–8 (!!!)	2	2	2	2	2
	Nuclei number at bulk of liquid	n_v_ 1/m^3^	1 × 10^5^ – 1 × 10^10^ (!!!)	1 × 10^6^ – 1 × 10^9^ (!!!)	6 × 10^6^ – 1 × 10^10^ (!!!)	1 × 10^7^	2 × 10^7^	8 × 10^6^	(1.5–2)10^7^	(8–10)10^6^
	Growth kinetic coefficient	a_3_ ms^−1^K^−3^	1.5 × 10^–8^ – 1.49 × 10^−5^ (!!)	1 × 10^–12^ – 2 × 10^−5^ (!!!)	8 × 10^–9^ – 6.5 × 10^−5^ (!!!)	1.5 × 10^–7^	2 × 10^–10^	1 × 10^–9^	(1–5)10^−10^	(1–3)10^–9^

**Table 7:** The tendency of the CAFE-3D model parameters influence.

Mould Type	Structure Parameter	Model Parametr				
		λ_mould_↑	α_cast-Ch_ ↑	ΔT_v_ ↑	n_v_ ↑	a_3_ ↑
Q-Q	d_av_	↑↓	-	↑	↓	↑
	CET	↑	-	↑	↓	↑
Q-Ch	d_av._	↑	↓	↑	↓	↑
	CET	↓	↑	↑	↓	↑
	γ	↑↓	↓	-	-	↓
HI-Ch	d_av_	↑↓	↓	↑	↓	↑
	CET	↓	↑	↑	↓	↑
	γ	↑↓	↓	-	-	↓
↑↓—no influence, ↑—value increasing, ↓—value decreasing, —not tested						

## Data Availability

The data presented in this study are available on request from corresponding author.
